# Multi-Faceted Roles of Stress Granules in Viral Infection

**DOI:** 10.3390/microorganisms13071434

**Published:** 2025-06-20

**Authors:** Ruihan Zhao, Xiangdong Li

**Affiliations:** 1 Jiangsu Co-Innovation Center for Prevention and Control of Important Animal Infectious Diseases and Zoonoses, College of Veterinary Medicine, Yangzhou University, Yangzhou 225009, China; zrh_keira97@163.com; 2 Joint International Research Laboratory of Agriculture and Agri-Product Safety, The Ministry of Education of China, Yangzhou University, Yangzhou 225009, China; 3 Key Laboratory of Protection & Utilization of Biological Resources in Tarim Basin, College of Life Sciences, Tarim University, Alar 843300, China

**Keywords:** stress granules, viral infection, innate immunity, phase separation, antiviral defense, viral countermeasures

## Abstract

Stress granules (SG), dynamic cytoplasmic condensates formed via liquid-liquid phase separation (LLPS), serve as a critical hub for cellular stress adaptation and antiviral defense. By halting non-essential translation and sequestering viral RNA, SG restrict viral replication through multiple mechanisms, including PKR-eIF2α signaling, recruitment of antiviral proteins, and spatial isolation of viral components. However, viruses have evolved sophisticated strategies to subvert SG-mediated defenses, including proteolytic cleavage of SG nucleators, sequestration of core proteins into viral replication complexes, and modulation of stress-responsive pathways. This review highlights the dual roles of SG as both antiviral sentinels and targets of viral manipulation, emphasizing their interplay with innate immunity, autophagy, and apoptosis. Furthermore, viruses exploit SG heterogeneity and crosstalk with RNA granules like processing bodies (P-bodies, PB) to evade host defenses, while viral inclusion bodies (IBs) recruit SG components to create proviral microenvironments. Future research directions include elucidating spatiotemporal SG dynamics in vivo, dissecting compositional heterogeneity, and leveraging advanced technologies to unravel context-specific host-pathogen conflicts. This review about viruses and SG formation helps better understand the virus-host interaction and game process to develop new drug targets. Understanding these mechanisms not only advances virology but also informs innovative strategies to address immune escape mechanisms in viral infections.

## 1. Introduction

Stress granules (SG) are dynamic, membraneless organelles formed in eukaryotic cells in response to various external stresses, such as viral infection, oxidative stress, or heat shock [1]. They function by halting non-essential protein translation and aggregating untranslated mRNA, translation initiation factors, and RNA-binding proteins (RBPs) in the cytoplasm [2]. The formation of SG relies on liquid-liquid phase separation (LLPS), with core regulatory factors including Ras-GTPase-activating protein-binding proteins (G3BP1/2) and T-cell intracellular antigen-1 (TIA1) [3,4]. As a critical component of the host innate immune response, SG exert potent antiviral functions by inhibiting viral protein synthesis, sequestering viral RNA, recruiting antiviral proteins (e.g., PKR, RIG-I), and disrupting viral replication cycles [5,6]. However, viruses have evolved diverse strategies to counteract SG formation, enabling them to evade host defenses and facilitate their own replication. Multiple studies on virus-SG interactions have unveiled complex molecular mechanisms, ranging from direct targeting of SG core proteins to modulation of host translation pathways. This review systematically summarizes the molecular mechanisms underlying SG formation, the diverse strategies employed by viruses to manipulate SG, and the implications for viral pathogenesis. Furthermore, we discuss future research directions in this frontier field.

## 2. Stress Granules: Fundamental Concepts

SG are dynamic condensates composed of translationally stalled mRNA, RBPs, and translation initiation factors, functioning in mRNA triage, translational control, and cellular stress adaptation [7]. SG assembly occurs through a dynamic multistage process driven by phase separation of scaffold proteins and multivalent RNA interactions. The process initiates with global translational suppression under stress conditions, leading to cytoplasmic accumulation of untranslated mRNPs [7]. G3BP1 oligomerizes via its NTF2L domain and binds RNA through RGG/RRM motifs, thereby driving phase separation of mRNA-RBPs complexes to form SG nucleation cores [3,8]. This phase separation capacity underlies the dynamic SG remodeling observed during viral infections, reflecting an evolutionary battleground between host defense and viral countermeasures [9]. By regulating mRNA metabolism, translational reprogramming, and stress signaling, SG formation constitutes a critical adaptive mechanism for cellular survival under viral challenge and diverse stressors.

### 2.1. Structure and Composition of SG

**Figure 1:**
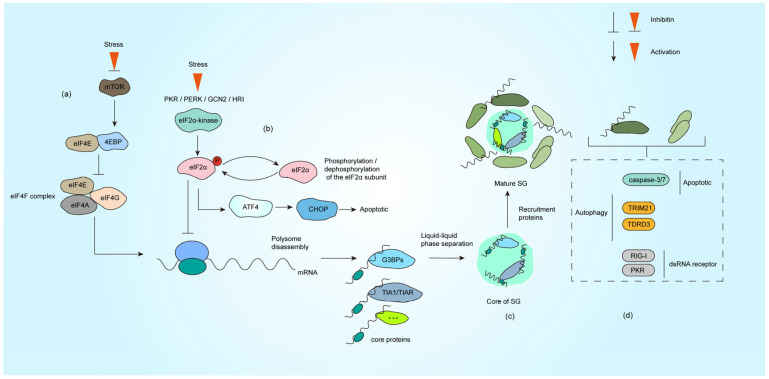
Formation process of SG under stress. (**a**) The impaired formation of the eIF4F complex under stress conditions leads to inhibition of translation initiation. (**b**) The activation of PKR, PERK, HRI and GCN2 under stress causes eIF2α phosphorylation, translation initiation is halted subsequent to the incorporation of phosphorylated eIF2α into the initiation complex. (**c**) SG nucleating proteins (such as G3BP1, TIA1, etc.) bind to untranslated mRNPs. These proteins nucleate the core of SG through LLPS, which subsequently recruit additional functional proteins (primarily RNA-binding proteins) to form mature SG. (**d**) Representative proteins involved in autophagy, apoptosis, and dsRNA sensing that are recruited to SG, with comprehensive descriptions provided in Section 3.1, Section 3.2, Section 3.3 and Section 3.4.

SG assemble dynamically through multivalent interactions and phase separation of core components. Proteomic analyses have identified over 300 SG-associated proteins, yet genetic studies by Yang et al. revealed G3BP1/2 as the most essential homologous proteins for SG nucleation [3,10,11]. G3BP1 employs its RNA recognition motif (RRM) and glycine-rich RGG domain to bind RNA, forming polymerization networks that recruit other SG components (e.g., TIA-1, eIF3) and drive LLPS [3,12,13]. The activity of G3BP1 is tightly regulated by post-translational modifications: acetylation (K376) weakens RNA binding to promote SG disassembly [14], while phosphorylation (Ser149) modulates assembly dynamics [15]. Moreover, TRIM21-mediated K63-linked ubiquitination of G3BP1 facilitates SG clearance via autophagy receptors SQSTM1/CALCOCO2, maintaining protein homeostasis (Figure 1d) [16].

Other core proteins contribute synergistically: TIA-1/TIAR proteins utilize low-complexity domains (LCDs) to induce phase separation, with tandem RNA-binding motifs enhancing nucleation efficiency [4,17]. eIF3, a translation initiation complex subunit, marks stalled ribosomes and integrates 48S preinitiation complexes (PICs) into SG networks [12,18]. Intriguingly, UBAP2L acts as an upstream nucleator, forming G3BP1-independent SG substructures and modulating heterogeneity through competitive interactions [19]. The functional interplay and post-translational regulation of these components collectively determine SG assembly kinetics, stability, and antiviral efficacy.

SG also incorporates auxiliary elements that fine-tune their dynamics: (i) Non-coding RNA: AU-rich elements in the 3′ UTRs of mRNAs and lncRNA are selectively sequestered for storage or translational suppression, while viral RNA may hijack SG functions [20,21]. (ii) Regulatory enzymes: Kinases (PKR, PERK) phosphorylate eIF2α to stall translation and promote SG assembly, counterbalanced by phosphatases PP1/PP2A that dissolve SG [7,22]. As a deacetylase, histone deacetylase 6 (HDAC6) regulates SG formation by interacting with G3BP1 and regulates post-translational modification of viral proteins [23,24]. Ubiquitin protease TRIM25 enhances antiviral responses by targeting viral RNA [25]. In addition to this, IRE1α acts as an ER stress sensor and co-condenses with SG via phase separation to amplify unfolded protein responses [26]. (iii) Metabolic modulators: NAD+-dependent sirtuins (e.g., SIRT1) regulate SG dynamics by deacetylating RBPs like TIA-1. Low NAD+ levels impair deacetylation, causing aberrant SG stabilization, while NAD+ repletion restores reversible disassembly [10]. This multilayered network ensures precise spatiotemporal control of SG formation, functional plasticity, and stress adaptation.

### 2.2. Mechanisms of SG Formation

Translational suppression is a prerequisite for SG assembly. Under stress conditions, cells activate the integrated stress response (ISR) to globally inhibit translation while selectively synthesizing critical proteins such as heat shock chaperones [27]. This strategy not only conserves cellular resources but also prevents the translation of potentially harmful mRNA (e.g., viral RNA or cancer-associated transcripts) [28]. The accumulation of untranslated mRNPs recruits SG core proteins like G3BP1, which drive phase separation to initiate SG assembly.

In eukaryotes, translational control primarily targets the cap-dependent initiation complex [7]. Stress-induced translation inhibition occurs via two distinct pathways: eIF2α-dependent or eIF2α-independent mechanisms [29]. The canonical translation process begins with eIF4E binding to the 5′ 7-methylguanosine mRNA cap, a step regulated by 4EBP. Under stress, dephosphorylated 4EBP competitively blocks eIF4E recruitment into the EIF4F complex, whereas phosphorylated 4EBP releases eIF4E to resume translation [30] (Figure 1a). Concurrently, phosphorylation of the eIF2α subunit disrupts the eIF2-GTP-Met-tRNAMet ternary complex, impairing 43S preinitiation complex assembly. Four eIF2α kinases (PKR, PERK, HRI, and GCN2) are activated by distinct stressors: PKR senses viral double-stranded RNA (dsRNA) [5], PERK responds to ER stress [31], HRI detects oxidative stress/heme deprivation [32], and GCN2 monitors nutrient deprivation [33] (Figure 1b). Viral infections often activate PKR directly via genomic RNA or indirectly through stress cascades involving other kinases.

mRNA plays a central role in SG dynamics. Polyadenylated mRNA and RNA-binding proteins form the structural backbone of SG [34]. Notably, nuclear export of newly synthesized cytoplasmic mRNA is critical for SG formation. Inhibition of mRNA transcription, splicing, or export reduces canonical SG assembly independently of eIF2α phosphorylation [35]. Additionally, viral RNA activates the pattern recognition receptor PKR, triggering eIF2α phosphorylation and SG induction, highlighting a key viral-host battleground [36,37].

SG assembly is currently viewed as a two-phase process: Nucleation and Maturation. During early stress (e.g., within 15 min of arsenite exposure), G3BP1 binds poly(A)-tailed mRNA, translation initiation factors (e.g., PABP1, eIF4G), and forms primary condensates. These structures exhibit irregular morphologies under microscopy, indicative of nascent organizational complexity.

Under the action of LLPS, SG further recruits a variety of proteins and develops into structures with functional partitions [18,38]. Studies reveal a dense core region (enriched with G3BP1-Caprin1-USP10 complexes) and a dynamic periphery (containing ATPases like DDX6), supporting a phase separation-mediated compartmentalization model [39,40]. This spatial organization allows SG to dynamically adapt to cellular conditions while maintaining functional specificity (Figure 1c).

## 3. Antiviral Functions of SG and Viral Countermeasures

SG exhibits diverse biological functions in cellular stress responses. In antiviral immunity, SG plays dual roles: on the one hand, they restrict viral infection by sequestering viral RNA, suppressing viral protein translation, and extensively participating in biological processes such as apoptosis, innate immunity responses, and autophagy. On the other hand, viruses can hijack the dynamic assembly and disassembly of SG to promote their own replication. This interplay between viruses and host SG underscores the critical importance of SG in antiviral defense mechanisms.

### 3.1. SG Antiviral Defense via Viral RNA Sequestration

SG functions as critical antiviral platforms by sequestering untranslated viral RNA, thereby disrupting viral replication cycles. During infection, viral RNA is recognized as foreign antigen by host sensors, leading to SG assembly and the aggregation of stalled translation preinitiation complexes [41]. These condensates physically isolate viral RNA from ribosomes, preventing their translation and replication. For example, G3BP1, a core SG nucleator, directly binds to the 3′ untranslated region (UTR) of enterovirus D68 (EV-D68) RNA, forming translationally silent ribonucleoprotein complexes that inhibit viral replication [42].

dsRNA interacts with SG components like G3BP1 and poly(A)-binding protein (PABP1), sequestering viral transcripts into SG and suppressing HBV transcription [43]. In alphavirus infections, SG formation limits viral replication by trapping viral genomic RNA, thereby reducing access to translation machinery [44]. These studies highlight a conserved antiviral mechanism where SG act as RNA sinks, disrupting viral replication through spatial and functional isolation of viral nucleic acids [34,45].

As an important signaling molecule for SG to exert antiviral effects, PKR plays a vital role in viral infection: it senses viral dsRNA and triggers SG assembly via phosphorylation of eIF2α. Upon activation, PKR stalls global translation and promotes SG formation, creating a hostile environment for viral replication [46]. For instance, herpes simplex virus 2 (HSV-2) mutants lacking the PKR antagonist ICP34.5 exhibit enhanced eIF2α phosphorylation, leading to SG assembly and attenuated replication [47] (Figure 1d).

### 3.2. Stress Granule Formation and Innate Immune Signaling Pathways

The interplay between SG and innate immunity has been extensively documented. Emerging evidence demonstrates that SG orchestrates multilayered antiviral defense by integrating and amplifying innate immune signaling cascades. Functioning as viral RNA enrichment platforms, SG directly interface with RIG-I-like receptor (RLR) pathway activation (Figure 1d) [48]. During SG assembly, viral RNA simultaneously accumulates with RLR signaling components (e.g., RIG-I, MAVS), enhancing viral sensing efficiency. For instance, Newcastle disease virus (NDV) infection triggers SG-mediated sequestration of viral positive-strand RNA and uncapped transcripts, which promotes RIG-I oligomerization and strengthens its interaction with mitochondrial antiviral-signaling protein (MAVS), ultimately amplifying type I interferon production [49,50].

Core SG protein G3BP1 further reinforces antiviral responses by forming a functional interactome with RLR pathway members, thereby optimizing viral RNA detection [48]. Paradoxically, G3BP1/2 also safeguards against RLR signaling hyperactivation, acting as rheostats to balance antiviral defense and immune homeostasis. This dual regulatory role prevents pathological inflammation while maintaining effective viral clearance [41]. Collectively, SG emerge as dynamic hubs that spatially coordinate innate immune activation, ensuring context-appropriate antiviral responses.

### 3.3. Synergistic Role of SG and Autophagy in Viral Infection

Autophagy is a conserved lysosomal degradation pathway in eukaryotes that restricts viral replication by selectively eliminating viral components (e.g., proteins, nucleic acids, replication complexes) while modulating type I interferon signaling [51]. As two critical quality control systems, stress SG and autophagy collaboratively regulate viral propagation through interconnected signaling networks during infection. Recent studies reveal that SG-autophagy crosstalk not only impacts viral replication efficiency but also fine-tunes host antiviral immunity [52,53,54,55]. For example, the E3 ubiquitin ligase TRIM21 suppresses oxidative stress-induced SG assembly by catalyzing K63-linked ubiquitination of G3BP1, while autophagy receptors SQSTM1/p62 and CALCOCO2/NDP52 recognize ubiquitinated G3BP1 to target SG for autophagic degradation [16]. In addition, the SG component TDRD3 (Figure 1d) acts as a selective autophagy receptor and affects SG clearance and cell survival by regulating the interaction of autophagosomes with LC3 [52].

Viruses exploit the SG-autophagy equilibrium to enhance replication. Coxsackievirus A16 infection triggers selective autophagic degradation of HDAC6, leading to SG disassembly and suppression of type I interferon responses, thereby promoting viral replication [56]. The ER stress pathway further links autophagy and SG dynamics. While the PERK-eIF2α axis typically drives SG formation, African swine fever virus hijacks this pathway via its K205R protein, activating ER stress-autophagy signaling to suppress antiviral immunity and repurpose SG components for viral replication factories [53]. Conversely, enteroviruses disrupt SG-autophagy cross-talk by cleaving adaptor proteins. The SG component TDRD3 acts as a selective autophagy receptor and affects SG clearance and cell survival by regulating the interaction of autophagosomes with LC3. However, Enteroviral 2A protease specifically targets TDRD3, blocking SG component recruitment to autophagosomes to evade host defenses and accelerate viral egress [52].

### 3.4. SG and Apoptotic Signaling Pathways

The dynamic equilibrium between SG and apoptosis serves as a critical checkpoint for viral replication. SG suppress cell death by sequestering key apoptotic effectors such as caspase-3/7 (Figure 1d). Structural studies reveal that caspase-3/7 are selectively enriched in SG through conserved motifs in their catalytic domains, where spatial confinement inactivates their enzymatic activity and blocks apoptotic cascades. Dissolution of SG abolishes this cytoprotective effect, confirming their functional role in apoptosis suppression [57]. Concurrently, virus-induced ER stress activates the PERK-eIF2α axis, which not only triggers SG formation via translational arrest but also initiates apoptosis through the ATF4-CHOP pathway [58].

Viruses exploit the interaction of SG and apoptosis for immune evasion. Human T-cell leukemia virus type 1 (HTLV-1) Tax protein binds deubiquitinase USP10 to suppress arsenite-induced SG assembly while enhancing ROS-dependent apoptosis, thereby promoting oncogenesis [59]. During NDV infection, RIP1 kinase is recruited to SG and anchors phosphorylated MLKL within these condensates, preventing necroptotic membrane pore formation [60]. Notably, viral regulation of the SG-apoptosis axis exhibits spatiotemporal specificity: West Nile virus (WNV) upregulates glutathione (GSH) via the Nrf2/ATF4 pathway early in infection to inhibit SG formation and sustain viral translation, while permitting late-phase SG assembly to mitigate excessive stress [61]. Conversely, in hepatitis C virus (HCV) persistence, NS5A stabilizes G3BP1 to promote SG formation, but excessive SG accumulation paradoxically activates ER stress-mediated apoptosis, creating a self-limiting viral replication cycle [62].

### 3.5. Viral Suppression of SG Formation via Different Strategies

**Figure 2:**
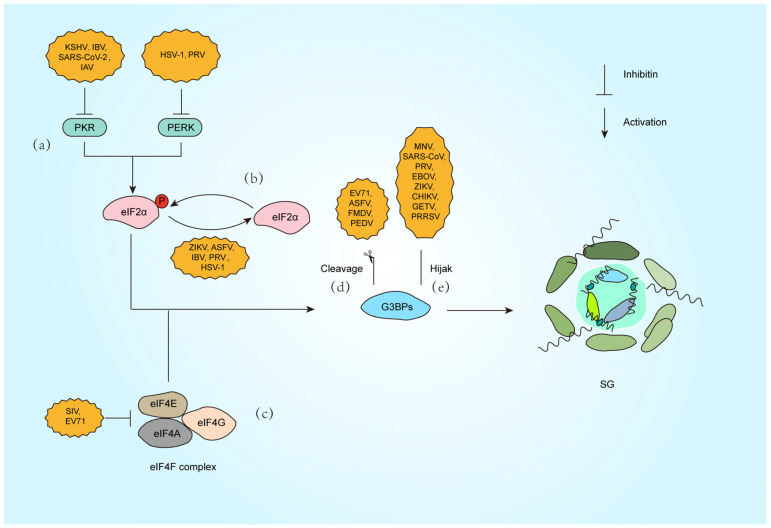
Strategies against SG formation by different viral infections. (**a**) Inactivate PKR (KSHV [63], IBV [64], SARS-CoV-2 [65], IAV [66]) or PERK (HSV-1 [67], PRV [68]). (**b**) Dephosphorylation of the eIF2α subunit (ZIKV [69], ASFV [70], IBV [71], PRV [72], HSV-1 [73]). (**c**) Inhibition of eIF4F complex (SIV [74], EV71 [75]). (**d**) Cleavage of G3BP1 (EV71 [76], ASFV [77], FMDV [78], PEDV [79]). (**e**) Hijack G3BP1 (MNV [80], SARS-CoV-2 [81], PRV [65], EBOV [82], ZIKV [83], CHIKV [84], GETV [85], PRRSV [86]).

As mentioned earlier in Section 3.1, PKR functions to restrict viral replication. To counteract PKR-mediated defenses, viruses deploy sophisticated strategies. Kaposi’s sarcoma-associated herpesvirus (KSHV) encodes ORF57, which binds PKR and prevents its autophosphorylation, effectively blocking SG formation and enhancing viral replication [48]. SARS-CoV-2 nucleocapsid (N) protein disrupts PKR activation by sequestering G3BP1, a PKR cofactor, thereby inhibiting SG assembly and promoting viral proliferation [49] (Figure 2a). Additionally, pseudorabies virus (PRV) induces dephosphorylation of eIF2α via the IE180 protein, bypassing PKR-dependent SG formation to sustain viral protein synthesis [50] (Figure 2b). These examples underscore the centrality of PKR in antiviral signaling and illustrate how viral targeting of PKR disrupts SG-mediated defenses.

Beyond PKR-eIF2α signaling, viruses exploit alternative pathways to modulate SG dynamics. Picornaviruses, such as enterovirus 71 (EV71), encode the 2A protease, which cleaves eIF4G to disrupt canonical SG assembly. This cleavage generates atypical SG that selectively sequester host mRNA while sparing viral transcripts, ensuring preferential translation of viral proteins [51,52] (Figure 2c). Similarly, PKR activation during influenza A virus (IAV) infection induces SG that restrict viral mRNA translation, but the virus employs the PA-X protein to degrade cytoplasmic poly(A) RNA and redistribute SG components like PABP1 into the nucleus and blocking SG nucleation independently of eIF2α phosphorylation. Notably, IAV also inactivates PKR via NS1 to inhibit eIF2α-dependent SG formation, showing the multiple regulatory effects of the same virus on SG formation [53] (Figure 2a).

Another example is porcine reproductive and respiratory syndrome virus (PRRSV), which recruits SG proteins such as G3BP1 to viral replication complexes. This interaction redirects SG components to support viral replication while suppressing host translation, demonstrating a strategy independent of eIF2α regulation [54]. Furthermore, Zika virus inhibits eIF2α phosphorylation but still blocks SG assembly by destabilizing G3BP1 aggregates, highlighting a multifaceted approach to evade translational arrest [55]. These mechanisms reveal how viruses bypass canonical stress pathways, rewiring translation machinery to favor their replication.

Beyond indirect modulation of SG dynamics, viruses directly disrupt SG assembly by hijacking core scaffolding proteins (Figure 2d,e). G3BP1, the central nucleator of SG, not only drives SG condensation but also exerts potent antiviral functions. Studies demonstrate that G3BP1 restricts viral replication through multiple mechanisms, including direct binding to viral dsRNA and amplification of RIG-I-mediated IFN-I production, thereby suppressing RNA virus propagation [87].

To circumvent this defense, diverse viruses have evolved proteolytic strategies targeting G3BP1. Enterovirus 71 (EV71) employs its 3C protease to cleave G3BP1 during late infection, dismantling SG to enhance viral replication [76]. Similarly, foot-and-mouth disease virus (FMDV) L protease (Lpro) selectively cleaves G3BP1/G3BP2 at specific sites, blocking SG nucleation without affecting the PKR-eIF2α phosphorylation pathway, highlighting precise SG-specific targeting [78]. African swine fever virus (ASFV) utilizes its cysteine protease pS273R to cleave G3BP1 at the G140-F141 site, generating truncated fragments incapable of SG nucleation and significantly boosting viral replication [77]. Notably, porcine epidemic diarrhea virus (PEDV) exploits host caspase-8 to mediate G3BP1 cleavage, revealing a novel indirect mechanism where viral activation of cellular proteases subverts SG assembly [79] (Figure 2d).

Viruses also suppress SG by spatially sequestering G3BP1. During murine norovirus (MNV) infection, G3BP1 is redirected to viral replication complexes, preventing SG nucleation. Intriguingly, G3BP1 knockout reduces MNV replication, suggesting dual exploitation: viral hijacking of G3BP1’s pro-replicative functions while neutralizing its antiviral role [80]. SARS-CoV-2 nucleocapsid (N) protein binds G3BP1 via its NTD-NTF2L domain interaction, trapping G3BP1 in viral ribonucleoprotein complexes to block SG formation and enhance genome packaging [81]. Our recent work reveals that PRV IE180 protein interacts with G3BP1/2 through its ICP4L-N domain, sequestering these nucleators in the nucleus to deplete cytoplasmic SG assembly capacity, ultimately promoting viral replication [65] (Figure 2e).

Alphaviruses inhibit host SG formation by targeting the SG core protein G3BP1, thereby promoting viral replication (Figure 2e). The ADP-ribosylhydrolase activity of alphavirus nonstructural proteins nsP3 dynamically regulates SG composition and disassembly by modulating post-translational modifications of host proteins, further suppressing antiviral responses [88]. In Chikungunya virus (CHIKV) infection, nsP3 recruits G3BP1 to viral replication complexes via its C-terminal FGDF motif binding to the NTF2-like domain of G3BP1, blocking SG assembly and enhancing viral RNA translation [84]. For Getah virus (GETV), the HDV domain of nsP3 directly disrupts the scaffolding function of G3BP1 by interacting with its NTF2-like domain, thereby impeding SG formation [85]. The NTF2-like and RGG domains of G3BP1 are critical for clustering alphavirus replication complexes and recruiting ribosomes, as their absence significantly diminishes viral replication efficiency. However, studies also show that G3BP1 does not affect replication in certain alphaviruses (e.g., GETV) [85,89].

## 4. Stress Granule Crosstalk with Other Granules in Viral Infection

RNA granules are dynamic cytoplasmic structures formed through phase separation of RNA molecules and proteins, widely present in eukaryotes, including SG and P-bodies. These granules regulate post-transcriptional gene expression (e.g., mRNA translation, storage, transport, and degradation), participating in cellular metabolism, stress responses, and developmental processes [90]. During viral infection, SG and PB form a cooperative antiviral defense: SG suppress viral replication by sequestering viral RNA and translation factors, while PB degrade viral Mrna [91,92]. Both structures dynamically assemble via phase separation and complementarily regulate RNA metabolism [93]. However, viruses have evolved dual strategies to dismantle this network. Viral IBs, such as the N/P protein condensates of respiratory syncytial virus (RSV) and Ebola virus, not only serve as replication factories but also inhibit SG assembly and reprogram host RNA metabolism by sequestering key SG proteins (e.g., TIA-1, G3BP1) or signaling molecules [94]. As dynamic membraneless structures, the compositional interplay among SG, P-bodies, and IBs reflects a broader battleground between host defense mechanisms and viral invasion.

### 4.1. PB and SG: Cooperative Antiviral Defense

SG and PB, two key cytoplasmic RNA condensates, exhibit both functional synergy and shared targeting by viral proteins during infection. SG enriched in RNA-binding proteins like G3BP1 and TIA-1 contrast with PB, which house RNA decay machinery (e.g., XRN1, CCR4-NOT complex) and GW182-family proteins. Despite partial spatial and compositional overlap, their roles are complementary: SG respond acutely to translational arrest and rapidly disassemble upon stress relief, whereas PB exhibit lower dynamics and mediate terminal mRNA metabolism [95,96]. This divergence underscores their distinct yet coordinated antiviral functions.

**Figure 3:**
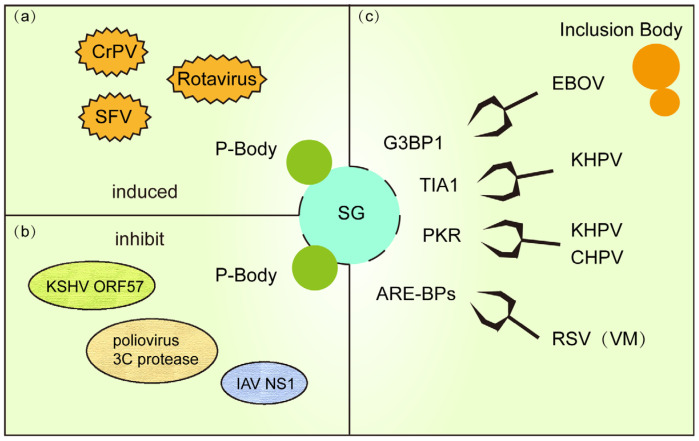
(**a**) PB and SG are co-formed in response to viral replication during viral infection. (**b**) Some viral proteins use a strategy of killing two birds with one stone to inhibit PB and SG formation. (**c**) IB (or VM) formed during viral infection hinders SG formation by stealing and sequestering important proteins for SG formation.

Viral infections often trigger cooperative SG-PB defense networks (Figure 3a). For example, early rotavirus infection induces SG (marked by G3BP1) and PB (marked by DCP1a) assembly to suppress viral protein synthesis by isolating viral RNA or restricting their translation [97]. Similarly, in Cricket paralysis virus (CrPV)-infected Drosophila cells, SG block viral translation initiation while PB degrade viral RNA, synergistically curbing infection [98]. During SFV infection, eIF2α phosphorylation-driven SG formation collaborates with PB-mediated viral RNA decay to restrict replication [99]. These observations highlight a “dual-barrier” system where SG and PB deploy distinct mechanisms to impede viral propagation.

To evade host defenses, diverse viruses have evolved strategies to concurrently disrupt both SG and P-bodies (Figure 3b). For instance, KSHV encodes the RNA-binding protein ORF57, which directly interacts with PB core components GW182 and Ago2 to inhibit PB scaffolding while blocking PKR signaling required for SG assembly, thereby enhancing viral RNA translation [93]. Similarly, poliovirus 3C protease degrades PB-associated proteins DCP1a and Pan3 to cripple mRNA decay pathways, while simultaneously cleaving SG nucleator G3BP1 to dismantle both condensates [100]. Influenza virus NS1 protein further exemplifies this dual targeting by binding RAP55 to dysregulate SG-PB crosstalk, suppressing antiviral RNA storage and degradation [101]. These examples underscore the centrality of SG-PB interplay in host defense and reveal a viral “two birds with one stone” tactic to hijack RNA regulatory networks.

### 4.2. Viral Disruption of SG Formation via Inclusion Body Hijacking

Viral inclusion bodies (IBs) are dynamic, membraneless structures formed during infection, comprising viral proteins, host factors, and viral genomic RNA/DNA. These condensates serve as central hubs for viral replication and transcription. For example, RSV IBs assemble via LLPS driven by nucleoprotein (N) and phosphoprotein (P), which form multivalent interaction networks to recruit viral polymerases and host cofactors, optimizing viral RNA synthesis [102]. Similarly, human metapneumovirus (HMPV) IBs organize viral RNA synthesis machinery through cytoskeleton-mediated aggregation [103]. Beyond replication, IBs act as molecular traps by sequestering antiviral host proteins (e.g., SG components), creating a proviral microenvironment [94].

Viruses exploit IBs to actively suppress SG assembly (Figure 3c). In RSV infection, IBs sequester critical signaling proteins like phosphorylated p38 kinase and O-GlcNAc transferase (OGT), inhibiting MAPK-activated protein kinase 2 (MK2) activity and blocking SG nucleation. RSV infection establishes specialized viral factories (VMs) that reprogram host SG and P-bodies by selectively recruiting their components, such as AU-rich element-binding proteins (ARE-BPs), into these replication hubs. This molecular sieving not only neutralizes SG antiviral functions but also repurposes host RNA metabolism factors to enhance viral genome replication [97,104]. Ebola virus (EBOV) IBs co-opt SG markers (e.g., TIA-1, G3BP1) into “atypical SG-like granules” that lack translation arrest functionality, ensuring uninterrupted viral protein synthesis [82]. Concurrently, human parainfluenza virus 3 (HPIV3) further illustrates this strategy: its N-P protein complex shields viral RNA from sensors, preventing PKR-mediated eIF2α phosphorylation and SG formation. HPIV3 exploits nucleoprotein (N)-phosphoprotein (P) interactions to assemble IBs that suppress SG formation [105]. These IBs shield nascent viral RNA from SG-mediated antiviral surveillance, effectively subverting host defense mechanisms through spatial compartmentalization of viral replication.

Recent studies reveal that Chandipura virus (CHPV) IBs recruit host PKR and TIA-1 via phase separation to form proviral condensates. Depleting TIA-1 or PKR severely impairs viral transcription, demonstrating their unexpected proviral roles within IBs [106]. Collectively, these findings position viral IBs not merely as replication factories but as molecular pliers that reshape host SG dynamics to favor viral persistence.

## 5. Conclusions and Future Perspectives

The dynamic battle between SG and viruses highlights a key evolutionary conflict in host-pathogen interactions. SG act as vital antiviral hubs, halting non-essential protein production, trapping viral RNA, and boosting immune signals like the PKR-eIF2α and RIG-I/MAVS pathways. However, viruses have evolved clever tactics to dismantle SG. These include cutting apart core SG proteins like G3BP1 (using enzymes such as EV71 3Cpro and FMDV Lpro), hijacking SG components into their own replication factories (as seen with SARS-CoV-2 N protein and PRV IE180), or diverting them into viral inclusion bodies (e.g., in RSV and Ebola virus infections). Viruses also manipulate stress pathways like autophagy and apoptosis to disrupt SG. This constant tug-of-war shows how viruses exploit the very adaptability of SG to evade immune defenses and thrive.

Moving forward, critical research goals are to visualize how SG behave in real-time within living organisms during infection using advanced tools like live-cell imaging and spatial transcriptomics. We also need to understand how differences in SG composition affect their ability to fight viruses and define the roles played by less-studied SG parts and their chemical modifications (like phosphorylation or ubiquitination) in these battles. Unraveling these details is crucial not only for fundamental virus research but also for developing new treatments. These could aim to strengthen SG defenses or block the viruses’ strategies for disabling them, carefully balancing powerful antiviral effects with potential harm to the host cell.

## Data Availability

No new data were created or analyzed in this study. Data sharing is not applicable to this article.

## Abbreviations

The following abbreviations are used in this manuscript:

PRV	Pseudorabies virus
SG	Stress granules
PB	Processing bodies
IB	Inclusion bodies
G3BP1/2	Ras-GTPase-activating protein-binding proteins
TIA1	T-cell intracellular antigen-1
RBPs	RNA-binding proteins
LCDs	Low-complexity domains
PICs	Preinitiation complexes
ISR	Integrated stress response
dsRNA	double-stranded RNA
PABP1	poly(A)-binding protein
HSV	herpes simplex virus
KSHV	Kaposi’s sarcoma-associated herpesvirus
IAV	Influenza A virus
PRRSV	Porcine reproductive and respiratory syndrome virus
RLR	RIG-I-like receptor
NDV	Newcastle disease virus
HDAC6	Histone deacetylase 6
HTLV-1	Human T-cell leukemia virus type 1
MAVS	Mitochondrial antiviral-signaling protein
WNV	West Nile virus
HCV	Hepatitis C virus
EV71	Enterovirus 71
FMDV	Foot-and-mouth disease virus
ASFV	African swine fever virus
PEDV	Porcine epidemic diarrhea virus
MNV	Murine norovirus
CJHIKV	Chikungunya virus
GETV	Getah virus
RSV	Respiratory syncytial virus
